# The Bergen 4-day treatment for panic disorder: replication and implementation in a new clinic

**DOI:** 10.1186/s12888-022-04380-6

**Published:** 2022-11-23

**Authors:** Hanne Moe Iversen, Thorstein Olsen Eide, Mathea Harvold, Stian Solem, Gerd Kvale, Bjarne Hansen, Kristen Hagen

**Affiliations:** 1 Molde Hospital, Møre og Romsdal Hospital Trust, 6412 Molde, Norway; 2 Center for Crisis Psychology, Faculty of Psychology, University of Bergen, Bergen, Norway; 3 Bergen Center for Brain Plasticity, Haukeland University Hospital, Bergen, Norway; 4 Department of Psychology, Norwegian University of Science and Technology, Trondheim, Norway; 5 Department of Clinical Psychology, University of Bergen, Bergen, Norway; 6 Department of Mental Health, Norwegian University of Science and Technology, Trondheim, Norway

**Keywords:** Panic disorder, Intensive treatment, Exposure, B4DT, CBT

## Abstract

**Background:**

Bergen 4-day treatment (B4DT) is a concentrated exposure-based treatment (cET), where the patient receives concentrated, individually tailored cognitive behavioral therapy (CBT) during four consecutive days. Previous findings have indicated that B4DT could be a promising treatment for panic disorder (PD).

**Aim:**

The aim of the present study was to evaluate the implementation of B4DT for panic disorder with- and without agoraphobia, at a new clinic. This is the first replication study for B4DT on panic disorder.

**Method:**

Thirty consecutively recruited patients with PD were included in an open trial design. Assessment of symptoms of panic disorder were measured with Panic Disorder Severity Scale (PDSS), while symptoms of generalized anxiety were assessed by Generalized Anxiety Disorder-7 (GAD-7) and depressive symptoms by Patient Health Questionnaire (PHQ-9) pre-treatment, post-treatment and at 3-month follow-up. Treatment satisfaction was measured with Client Satisfaction Questionnaire (CSQ-8) post-treatment.

**Results:**

The results showed a significant reduction in symptom severity from pre-treatment to post-treatment (d = 4.32), and at 3-month follow-up (d = 4.91). The proportion of patients classified as fulfilling the criteria for remission was 80.0% at post-treatment and 86.7% at follow up. There was a significant reduction in symptoms of depression and generalized anxiety. Treatment satisfaction was high and none of the patients dropped out.

**Conclusion:**

The current study replicated the results from the original study and indicate that the treatment can be successfully implemented at new clinics. B4DT may be a promising treatment for panic disorder and comorbid symptoms of generalized anxiety and depression. Larger and more controlled studies are needed to establish the efficacy of B4DT for panic disorder.

## Background

Panic disorder (PD) is characterized by the presence or a fear of panic attacks, with rapid onset and intense anxiety. It includes symptoms like shaking, sweating and racing heart [1]. PD has a lifetime prevalence ranging up to 4% [2] and a median age of onset was 32 [3]. Studies have shown that PD is associated with comorbid anxiety disorders, mood disorders, and psychotic disorders [4, 5]. Patients with PD report reduced quality of life, poor sense of health, more frequent use of medical services and more marital issues [6].

Cognitive behavioral therapy (CBT) is regarded as a first-line treatment for panic disorder with or without agoraphobia [7, 8]. CBT based treatment for panic disorder have been found to be effective in a diversity of treatment formats. Brief, intensive or concentrated (BIC) treatment is characterized by prolonged therapy sessions over a very short period of time, but the total number of sessions is often relatively similar to standard CBT [9–11]. Concentrated CBT may have the advantage that it can make a positive change in a shorter timeframe [9, 12]. A recent meta-analysis of brief, intensive and concentrated (BIC) treatment for anxiety disorders in adolescents indicated that BIC had higher rate of response and remission, compared with standard CBT for a [13].

The Bergen 4-day treatment (B4DT) is a concentrated exposure treatment (cET), where the patient receives individually tailored CBT delivered during four consecutive days. B4DT can be categorized as an individual treatment delivered in a group setting, since the treatment is delivered in groups with 3 to 6 patients with a therapist-patient ratio of 1:1. The B4DT has proven highly acceptable for treating obsessive-compulsive disorder, with very low drop-out rates and the results show that almost 70% were classified as recovered at 12-month follow up [14]. A follow-up study demonstrated that the treatment outcome was stable 4 years after completing treatment [15].

There has only been published one study of B4DT for PD. The pilot study (*N* = 29) found promising results. The proportion of treatment responders was 72.4% at both post-treatment and at 3-month follow-up [16]. They also found very high treatment satisfaction, with a mean Client Satisfaction Questionaire-8 (CSQ-8 [17];) score of 30.2, where the maximum score is 32. Although the results are promising, there has not yet been demonstrated whether the results can be replicated or implemented at a new site with other clinicians.

The aim of this study was to examine if the promising effects from the pilot study on the B4DT for PD with or without agoraphobia is transferable to a new clinic. We hypothesized that there would be no significant differences between the current study and the previously published pilot study on B4DT for PD, based on previous studies on implementation of B4DT on OCD in new clinics [18, 19].

## Method

### Participants and procedure

**Fig. 1:**
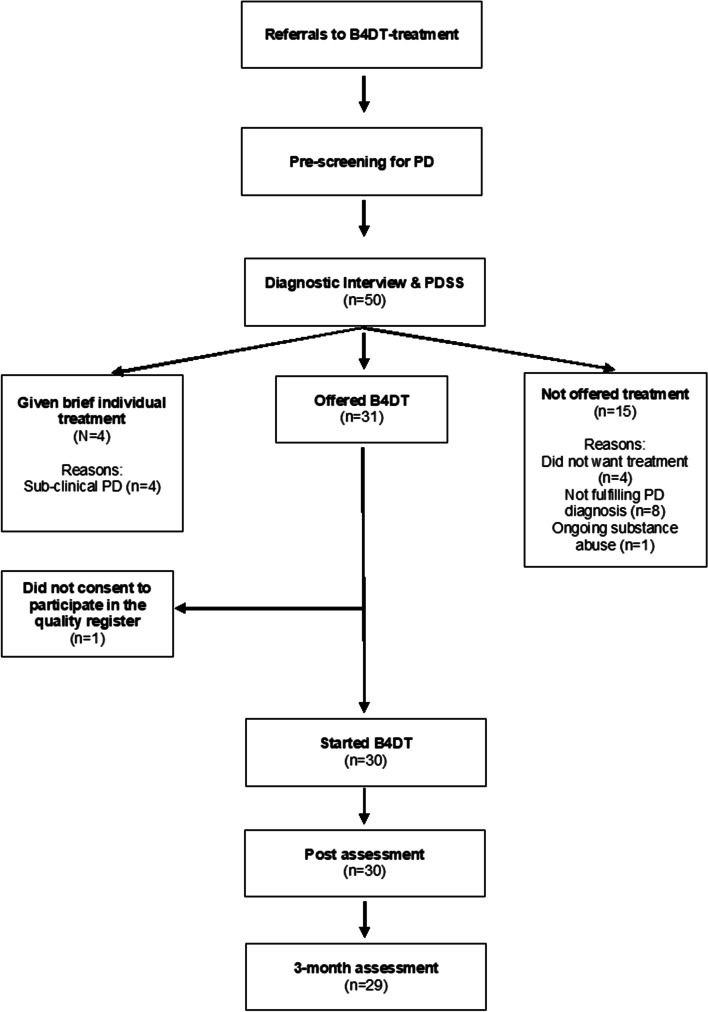
Flow chart

The study was conducted at an outpatient clinic in Molde, which is a part of the specialist health care in Helse Møre og Romsdal Hospital Trust in Middle Norway. The patients in the study were consecutively referred to their local outpatient specialist health care unit by their general practitioner, and if the condition were considered to grant them treatment in the specialized health care, the patients were referred to the anxiety clinic. All patients were assessed by an experienced therapist using the Mini International Neuropsychiatric Interview (MINI [20];). MINI is a short structured diagnostic interview used for screening axis-I DMS-IV disorders. Patients who fulfilled criteria for PD or agoraphobia were considered for participation. Patients were excluded if they did not speak Norwegian, were suicidal, or suffered from ongoing substance abuse, psychosis, or bipolar disorder (See Fig. 1 for Flow Chart). Most of the patients had symptoms of both panic disorder and agoraphobia (76.7%, *n* = 23). See Fig. 1 for patient flow in the study.

In total, 20 of the patients (66.7%) had a comorbid psychiatric disorder.. The comorbid disorders were as following: 56.7% (*n* = 17) had comorbid depression, 16.7% (*n* = 5) had GAD, 6.7% (*n* = 2) had PTSD, 3.3% (n = 1) had SAD, 3.3% (n = 1) had OCD, and 3.3% (n = 1) had health anxiety. Patients using pharmacotherapy were on medication before they were referred to the anxiety clinic and they had to be on a stable dose 4 weeks before starting the B4DT. Use of medication was registered at the initial interview. Of the patients, 18 (60.0%) were using psychotropic medication. Eleven (36.7%) used SSRI, eight (26.7%) used benzodiazepines, and one (3.3%) used medication for ADHD. Patients on SSRI (36.6%, *n* = 11) were informed to maintain the same medication doses prior to and during the treatment period. The patients who used benzodiazepines (26.7%, *n* = 8) were encouraged to not use these medications during the exposures or directly afterwards. All patients adhered to this instruction, but some used it in the evening to help them sleep. No withdrawal effects were reported by the patients.

There was a majority of female patients (70.0%, *n* = 21), and the mean age of the patients was 38.0 years (SD = 13.10, range = 21–64). The mean duration of PD was 13.43 (SD = 14.60) years, with a range from 1 to 48 years. None of the patients were in an acute phase, as all patients had suffered from panic attack for more than 3 months before starting treatment. The mean age of onset for panic disorder was 13.43 years (SD = 14.60, range = 3 months – 47 years). Six of the patients had suffered from PD for less than a year. See Table 1 for a summary of sample characteristics.

**Table 1:** Sample characteristics

	M (SD)/N(%)
Female gender	21 (70.0)
Age	38.00 (13.10)
Duration of the disorder (years)	13.43 (14.60)
Previous treatment	22 (73.3%)
In relationship	19 (63.3%)
University/College	9 (30.0%)
Working/student	17 (56.7%)
Comorbid disorders	20 (66.7%)
Psychotropic medication	18 (60.0%)
SSRIs	11 (36.7%)
Benzodiazepines	8 (26.7%)

The patients who met inclusion criteria were assigned to a treatment group, and requested to participate in a voluntarily, written consent-based quality register. All patients in the study agreed to take part of the quality register and none of the patients’ withdrew their consent. One patient did not agree to take part of the quality register, but were offered and completed treatment.

### Measures

Assessments were carried out pre- and post-treatment and at 3-month follow-up. Patients completed online self-report questionnaires concerning generalized anxiety, depression, and client satisfaction. If patients did not complete self-report questionnaires according to a pre-set time limit, they received a reminder in form of an automatic text message. An independent assessor, who were not involved in the treatment conducted PDSS interview at post-treatment and follow-up, while the pre-treatment interview was conducted by the therapist that did the assessment before treatment.

#### Panic disorder severity scale

(PDSS [21];) is an interview for assessing panic disorder severity, and is used as the primary outcome measure. The seven items are rated on a scale from 0 to 4. The maximum score of PDSS is 28. High scores indicate increased severity. For patients without agoraphobia, scores from 0 to 1 correspond to “Normal”, scores from 2 to 5 to “Borderline”, 6 to 9 to “Slightly ill”, 10–13 to “moderately ill”, and 16 and above is considered as “Markedly ill”. According to symptom reduction, a reduction in PDSS between 100 to 75% was considered as “Very much improved”, a reduction between 74 to 40% as “Much improved”, and a reduction between 39 to 10% was considered “Minimally improved” [22]. The Furukawa et al. [22] criteria defined response when the PDSS is reduced with at least 40% from pre-treatment to post-treatment. Remissions is accomplished when the post-treatment is also under the category “borderline ill”, which is 5 for PD and 7 for PD with agoraphobia. PDSS has previously showed good psychometrical properties [21].

#### Generalized anxiety disorder

(GAD-7 [23];) is a questionnaire for measuring symptoms of generalized anxiety disorder. The questionnaire contains seven items, and the total scores range from 0 to 21. Scores from 0 to 4 correspond to “Minimal anxiety”, 5 to 9 correspond to “Mild anxiety”, 10 to 14 correspond to moderate anxiety, while 15 to 21 correspond to “Severe anxiety” [23].

#### Patient health questionnaire 9

(PHQ-9 [24];) is a questionnaire for measuring depressive symptoms. It contains nine items, and each item is reported on a four-point Likert scale (0 = not at all, 3 = almost every day). The total scores range from 0 to 27, where 0 to 4 correspond to “None”, 5 to 9 correspond to “Mild”, 10 to 14 correspond to “Moderate”, 15 to 19 correspond to “Moderate-Severe, and 20 and above correspond to “Severe” [25].

#### The client satisfaction questionnaire

(CSQ-8) [17] is an eight-item questionnaire used to measure the patients’ degree of satisfaction with the treatment. The total score ranges from 8 to 32, and higher scores indicates higher level of treatment satisfaction [17].

### Therapists

The therapists (*n* = 8) were either clinical psychologist or psychiatrists. The two group leaders had eight and 10 years of experience. The therapists had attended at least three B4DT-groups, whereas at least one was on panic disorder.

The therapists worked as a team and had daily therapist meetings. The function of the meetings was to make sure that the therapists knew about the progress and challenges for each patient. The group leader would decide which therapist and patient that would work together in the next session and would supervise and assist if needed.

### Treatment

The B4DT for panic disorder [16] is inspired by the B4DT model for OCD [14, 26, 27], the cognitive model of PD [28] and the inhibitory learning approach to exposure therapy [29]. The B4DT treatment for panic disorder was delivered during four consecutive days and included a booster session 3 months after treatment (45 minutes). The treatment was delivered as individual treatment in a group setting, as the groups consisted of three to six patients, where the therapist-patient ratio was 1:1.

The main focus of the B4DT is the” LEaning in Technique” (LET), which is an approach for exposure that emphasizes that the patient attempt to actively shift from avoiding unpleasant bodily symptoms, thoughts and emotions, to instead actively approach whatever elicits relevant anxiety/discomfort and “lean” into the bodily symptoms, thoughts and emotions that are awoken in the exposure.

The content of the first day (3 hours) of treatment was psychoeducation about PD and the treatment. The information was delivered in a group setting, and the patients were introduced to each other and the therapists. The patients also prepared their individual exposure tasks, and the tasks were evaluated in the group setting. The first day of treatment was dedicated to psychoeducation about PD and the treatment. The second and third day (7 hours each) were dedicated to exposure and behavioral experiments based on a CBT rationale. The treatment involved both interceptive exposure that targets to the bodily sensations that each patient report that they fear, and in vivo exposure to the external situations where they experience fear or that they tend to avoid. The longer therapy sessions allowed sufficient time for the therapist to do exposure tasks together with the patients in a wide range of themes and settings that the patient reported as most challenging and/or important. The group met three times on day two and three to report back to the group and to share experiences. On the second and third day, exposure plans for the evening were made and the patients reported to their therapist with a SMS at the end of the evening. The patients were also told that they could call their therapist, if there was anything they struggled with. On the afternoon the third day, friends and family were invited to a psychoeducation meeting to learn more about the disorder and the treatment (1 hour).

The focus on the fourth day (3 hour) The focus on the last day was to teach the patients how to maintain and continue treatment effects.. The patient and therapist made a plan for continued exposure for the 3 weeks after treatment. The patients were encouraged to register the progress online each day of the first 3 weeks. They received no feedback from therapist during this period. Three months after the treatment, patients were offered an individual session for treatment consolidation. This session did not include exposure.

### Statistical analyses

The expectation-maximization method of SPSS, version 24, was used to implement missing values on the self-report scales (PHQ-9, GAD-7). The total amount of missing data was 5.0%. Little’s MCAR test indicated that the self-report data were missing completely at random, *χ*^2^(12) = 14.263, *p* = 0.284. This allowed the use of repeated measures ANOVA to investigate change in symptoms from pre- to post and follow-up. Repeated measures ANOVA for PDSS, GAD-7, and PHQ-9 were conducted using Greenhouse–Geisser corrections when Mauchly’s test of sphericity was significant. For post-hoc analysis, Bonferroni corrections were used. Cohen’s d*,* defined as (M pre-treatment – M post-treatment)/SD pre-treatment, was used to calculate effect sizes [30]. The results were compared with the results from the pilot B4DT for PD in Bergen [16] with a t-test for independent samples.

## Results

### Primary outcome measure

There were no dropouts from the treatment. All patients reported symptoms using the PDSS at pre-treatment and at post-treatment, while only one patient (3.3%) missed the PDSS interview at follow-up.

There was a significant reduction in symptoms of panic disorder over time, F_(1.591, 44.546)_ = 281.815, *p* < .001, partial eta squared = .910. The improvement from pre-treatment to post-treatment was significant (*p* < 0.001, d = 4.32), as was the improvement from pre-treatment to follow-up (*p =* 0.01. d = 4.91). There was also significant improvement from post-treatment to follow-up (*p* = 0.03, d = 0.43). See Table 2 for details on changes in symptoms following treatment. There were no significant differences with respect to treatment outcome between those who had suffered from PD for less than a year and those with longer illness duration, F_(1,63)_ = 1.344, *p* = .856.

**Table 2:** Changes on the primary and secondary outcome measures following treatment

	Pre	Post	Follow-up	d post	d follow-up
PDSS	19.83 (3.44)	4.37 (3.72)	2.62 (3.57)	4.32	4.91
GAD-7	12.70 (4.63)	5.72 (3.77)	4.96 (4.47)	1.65	1.70
PHQ-9	14.40 (6.50)	6.62 (5.82)	6.68 (6.48)	1.26	1.19

*PDDS* Panic Disorder Severity Scale, *GAD-7* Generalized Anxiety Disorder-7, *PHQ-9* Patient Health Questionnaire-9, d = Cohen’s d (M_pre_ – M_post_)/SD_pooled_)

Three repeated measures ANOVAs tested whether using pharmacotherapy was associated with treatment outcome. The first analysis compared treatment effects for patients using benzodiazepines and/or SSRIs (*n* = 15) with the remaining sample. There was no significant interaction effect, F_(1,57)_ = 0.938, *p* = .379. The second analysis investigated treatment effects for patients using SSRIs (*n* = 11), and found no significant interaction effect, F_(1,57)_ = 1.273, *p* = .284. The third analysis, investigated treatment effects for patients using benzodiazepines (*n* = 8), and found no significant interaction effect, F_(1,63)_ = 0.097, *p* = .870.

The response and remission rates based on Furukawa [22] criteria for PDSS were high. At post-treatment, 56.7% (*n* = 17) had “Very much improvement” (75–100% reduction), 40.0% (*n* = 12) had “Much improvement” (40–74%), while 3.3% (n = 1) had “Minimally improvement” (10–39%). At follow up, 83.3% (*n* = 25) had “Very much improvement”, 10% (*n* = 3) had “Much improvement”, 3.3% (n = 1) had “Minimally improvement” (10–39%), while 3.3% (n = 1) had missing data (see Table 3).

**Table 3:** Status at follow-up based on criteria from Furukawa et.al [22].

	Post-treatment (N/%)	Follow-up (N/%)
Very much improved (75–100%)	17 (56.7%)	25 (83.3%)
Much improved (40–74%)	12 (40.0%)	3 (10.0%)
Minimally improved (10–39%)	1 (3.3%)	1 (3.3%)
No improvement (0–10%)	0	0
Missing data	0	1 (3.3%)

### Secondary outcome measures

There was also a significant reduction in depressive symptoms over time, = F_(1.059, 30.699)_ = 42.632, *p* < .001, partial eta squared = .595. There was a significant reduction from pre-treatment to post-treatment (*p* < .001, *d* = 1.26), and from pre-treatment to follow-up (p < .001, d = 1.19), but not from post-treatment to follow-up (*p* = .435, d = 0.18). There was also a significant reduction in symptoms of generalized anxiety, F_(1.568,45.485)_ = 50.454, *p* < .001, partial eta squared = .635, *d* = 1.70. There was a significant reduction from pre-treatment to post-treatment (*p* < 0.01, d = 1.65), and from pre-treatment to follow-up (p < .001, d = 1.70), but not from post-treatment to follow-up (p = .435, d = 0.12). Patients scored high on treatment satisfaction, as indicated by a score of 30.71 (SD = 1.61) on the CSQ-8.

### Comparisons with the pilot study on B4DT for panic disorder

The current study had a significant higher pre-score on PDSS (p < .001, d = 1.09) compared to the pilot study on B4DT for PD at the original site, but there were no differences on post-treatment (*p* = .352, d = 0.24). However, the PDSS score at 3-month follow-up was significant lower (*p* = .23, d = 0.58). The current study had significant higher scores on pre PHQ-9 (*p* = .030, d = 0.58), but not at post-treatment (*p* = .941, d = 0.02). There was no significant difference is CSQ-8 score post-treatment (*p* = .341, d = 0.25). For details, see Table 4.

**Table 4:** Comparisons between original study and current replication study

	Original site	Current study	p	d
Pre PDSS	15.79 (3.97)	19.83 (3.44)	<.001	1.09
Post PDSS	5.34 (4.22)	4.37 (3.72)	.352	0.24
Follow-up PDSS	4.82 (3.65)	2.62 (3.57)	.023	0.61
Pre PHQ-9	10.79 (5.93)	14.40 (6.50)	.030	0.58
Post PHQ-9	6.72 (4.48)	6.62 (5.82)	.941	0.02
Follow-up PHQ-9	n.a.	6.68 (6.48)	–	–
Pre GAD-7	11.93 (3.76)	12.70 (4.63)	.487	0.18
Post GAD-7	6.13 (3.06)	5.72(3.77)	.649	0.12
Follow-up GAD-7	n.a.	4.96 (4.47)	–	–
CSQ-8 post	30.16 (2.68)	30.71 (1.61)	.341	0.25

*PDDS* Panic Disorder Severity Scale, *GAD-7* Generalized Anxiety Disorder-7, *PHQ-9* Patient Health Questionnaire-9, *CSQ-8* Client Satisfaction Questionaire-8, *d* Cohen’s d (M_pre_ – M_post_)/SD_pooled_)

### Comparisons with standard CBT for panic disorder

To benchmark the results from the current study to standard CBT for panic disorder with and without agoraphobia, we compared it to standard CBT studies that have used PDSS as outcome measure. This included nine studies with a total of 517 patients. For further details on the procedure, see [16].

The current study had a significant higher pre-score on PDSS (*p* < .001, d = 1.57) comparing the current study with standard CBT. Further, the current study had significantly lower scores on post-treatment (p < .001, d = 0.69) and at 3-month follow-up (*p* = .003, d = 0.60).

## Discussion

This is the first replication of the promising results from the pilot study. The results indicate that the B4DT can be replicated at other clinics using other therapists and still obtained the same promising results. This is in line with previous studies with implementing the B4DT-treatment for OCD to new sites [19, 31] and countries [32]. In the current study, we found that there were significantly higher PDSS scores before treatment, and significant lower scores at follow-up in the current study compared to the previous study on B4DT for PD. There were no significant differences between the studies at post-treatment. Comparing the current study with standard CBT, we found significantly higher scores on PDSS pre-treatment, but significantly lower scores at both post-treatment and follow-up.

There was a significant reduction in symptoms of PD from pre-treatment to post-treatment and from post-treatment to follow-up. This is in line with the findings from the original study [16]. This may imply that the patients continue to use the skills that they practiced in treatment. The secondary measures also showed a significant reduction in symptoms of anxiety (GAD-7) and depression (PHQ-9) from pre-treatment to post- and follow-up. This is in line with previous findings on B4DT for PD [16] and for B4DT treatment for OCD [14, 15, 18, 19, 27]. It should also be noted that all patients completed the treatment. This is in line with the findings from Hansen et al. [16]. The low dropout rate may be a result of the treatment’s concentrated format. This is interesting given previous findings that drop-out is found to be a challenge in exposure based treatment for PD [33].

The scores on CSQ-8 showed high levels of satisfaction with the treatment. The patients reported their experience of the length and the format of the treatment as positive; they reported feeling understood by the therapist and that the treatment was relevant for their problems. The patients would recommend the treatment to a friend with PD, and they would return to the clinic if needed.

There are several factors thought to be important for the promising results observed.

The concentrated format does not mean reducing the amount of therapy given. The format also gives an advantage to therapists as they have a lot of time together with the patient and have the possibility to do exposure exercises in several different settings that are most relevant for the patient. The concentrated format could also make it easier for the patient to focus and prioritize the treatment. In addition, the treatment is conducted within a group setting, so the patients have the possibility to share their experiences and learn from each other.

The study had some obvious limitations. The study was an open trial with no control condition. Since the treatment was delivered as a part of the public health service, the treatment was not videotaped, so the competence and adherence to the protocol was not evaluated. There was no evaluation of the amount of exposure tasks completed by patients during the follow-up period. Although the results suggested that use of SSRIs and/or benzodiazepines was not associated with treatment outcome, it remains unclear whether there could have been any potential late-onset gains of pharmacotherapy for some patients. The potential efficacy of the B4TD for PD should also be tested also in samples with acutely-ill and medication-free patients. Further, the effect sizes should be interpreted with caution because of the open label design (not including a control group), the lack of randomization of patient or therapists, and consequently no double-blinded assessments. This limits the generalization of the results. The comparisons with standard CBT are not straightforward because effect sizes could differ due to different study designs. The results are promising, but larger and controlled studies are needed to conclude about the treatment’s efficacy. Although the current study was conducted at a new clinic, the study should also be replicated in other sites and cultures.

## Conclusion

The study is the first replication of B4DT for panic disorder. The results indicates that the concentrated treatment format panic disorder is transferable and can be effective in treating panic disorder also at other sites using different therapists. The results indicated that the treatment is also associated with symptom reduction regarding comorbid symptoms of depression and generalized anxiety. This also strengthen previous findings that BIC treatment may be a valuable supplement to more standard formats of CBT. Larger and more controlled studies are needed.

## Data Availability

The dataset for the study is available from the first author upon reasonable request.

## Abbreviations

ANOVA: Analysis of variance
B4DT: Bergen 4-day treatment
BIC: Brief, intensive and concentrated treatment
CBT: cognitive behavioral therapy
cET: concentrated exposure-based treatment
GAD-7: Generalized anxiety disorder-7
MINI: Mini International Neuropsychiatric Interview
PHQ-9: Patient health questionnaire-9
